# The Relationship between Cognitive Performance and Quality of Life in Elite Athletes after Spinal Cord Injury

**DOI:** 10.3390/ijerph19020948

**Published:** 2022-01-15

**Authors:** Agata Goraczko, Alina Zurek, Maciej Lachowicz, Katarzyna Kujawa, Grzegorz Zurek

**Affiliations:** 1 Department of Biostructure, Wroclaw University of Health and Sport Sciences, 51-612 Wroclaw, Poland; agagoraczko@gmail.com (A.G.); maciej.lachowicz@ideis.pl (M.L.); katarzyna.kujawa@awf.wroc.pl (K.K.); 2 Clinic of Neurorehabilitation, 54-519 Wroclaw, Poland; 3 Institute of Psychology, University of Wroclaw, 50-527 Wroclaw, Poland; alina.zurek@uwr.edu.pl

**Keywords:** cognitive performance, cognitive disorders, spinal cord injury, elite athletes

## Abstract

Background: The present investigation was designed to determine cognitive performance and quality of life (QoL) in a group of elite athletes who sustained spinal cord injury (SCI). Methods: nine participants suffering a SCI participated in the study. Different cognitive functions were evaluated through the following tests: COWAT, Digit Span, Stroop color–word and QoL through the WHOQoL-BREF scale. Results: Generally, participants positively assessed their overall quality of life and health status. Although the tests conducted indicate reduced cognitive function among the athletes, it did not affect the reduction in QoL. Single correlations between the results of cognitive tests and QoL could be treated as coincidental. Conclusions: Despite the observed decline in selected cognitive functions, the participants positively assessed their quality of life and physical health.Reduced cognitive functioning could be influenced by the impact of sleep-disordered breathing, pain, depressive disorders and medication. This indicates the need for an individualized approach to define the patient’s deficits, needs and best care. Further studies with a larger group of participants are needed.

## 1. Introduction

In addition to the loss of motor and sensory function below the level of injury, spinal cord injury (SCI) is associated with a number of other consequences, which also typically include cognitive impairment. Cognitive functioning refers to, among other things, a person’s ability to process thoughts [1]. The term cognitive function mainly refers to areas related to thinking such as memory, ability to learn new information, speech and communication. The information processing pathway successively includes stimulus perception, selective attention, working memory and executive functions [2].

Data on the prevalence of cognitive deficits in patients with spinal cord injury vary due to the definition of cognitive deficits used and the procedures used to assess and measure them in a given study [1]. It is estimated that cognitive disorders (CD) affect between 30 and up to 60% of the adult population after an SCI [1,3,4]. The risk of CD in individuals after an SCI is 13 times higher than in individuals without such injury [3,4]. Dovler et al.’s (1997) study demonstrated distinct patterns of CD in individuals with chronic SCI which affected cognitive areas such as processing speed, new learning memory and executive functioning [5,6].

There are various causes to explain the occurrence of CD in individuals after an SCI. Often, spinal cord injury is accompanied by brain injury and/or an upright injury to the cervical spine (whiplash), which leads to brain dysfunction [4,7]. Respiratory problems during sleep are common in individuals with tetraplegia. Anoxia due to obstructive sleep apnea or shallow breathing leads to reduced neuropsychiatric function [8]. Decreased saturation at night affects concentration, verbal attention, cognitive flexibility, short-term and long-term memory [9]. A significant number of drugs, such as antispastic, analgesics, sleep aids and antidepressants, interferes with cognitive function [7,10]. In a study by Davidofi et al. (1992), depression was observed in 33% of people after a spinal cord injury, which reduces somatosensory input, thus reducing cortical arousal and cognitive performance [1]. A serious problem in people after a spinal cord injury is neuropathic pain, which is difficult to treat [11]. Chronic pain causes a decrease in the density of nerve tissue in the cerebral cortex, which is responsible for pain perception (a loss of about 0.5% per year), a decrease in cerebral flow (mainly in the thalamus and basal nuclei), which clinically manifests itself as behavioral changes and cognitive impairment [12]. Studies in mice have shown that extensive post-traumatic inflammation associated with SCI results in neuronal loss, cellular dysfunction through increased endoplasmic reticulum stress and impaired neurogenesis in areas associated with CD and depression [13].

Molina et al. (2021) demonstrated the occurrence of cognitive impairment in individuals after SCI already in the subacute phase, which worsened over time [14]. This may not only affect the disruption of the first, most intensive and most important stage of rehabilitation, but may also affect the individual’s quality of life and their eventual integration into society [14,15]. This is supported by the findings of Craig et al. (2017), who found that the development of negative mood states was a significant problem in individuals with cognitive impairment after their transition to the community, at a time when personal resources were severely limited [4]. There are many papers about the relationship between QoL and cognitive impairment in various disease entities, such as mild thyroid hormone deficiency, migraine, schizophrenia, cancer, multiple sclerosis and cardiac goiter, but a review of the literature shows—to our best knowledge—that there is a lack of research determining the relationship between cognitive impairment and quality of life in individuals after an SCI [16,17,18,19,20,21]. According to Badenhorst et al. (2018), the QoL of individuals with rugby-related SCI was higher than that of non-sporting participants of other studies [22]. Therefore, we hypothesize that a group of elite athletes after SCI are characterized by positive quality-of-life scores and cognitive functions above average and that there are correlations between them.

The purpose of this study is to evaluate cognitive functions in outstanding athletes after an SCI and to demonstrate whether there is a relationship between cognitive level and quality of life in this group of individuals.

## 2. Materials and Methods

### 2.1. Participants

**Figure 1:**
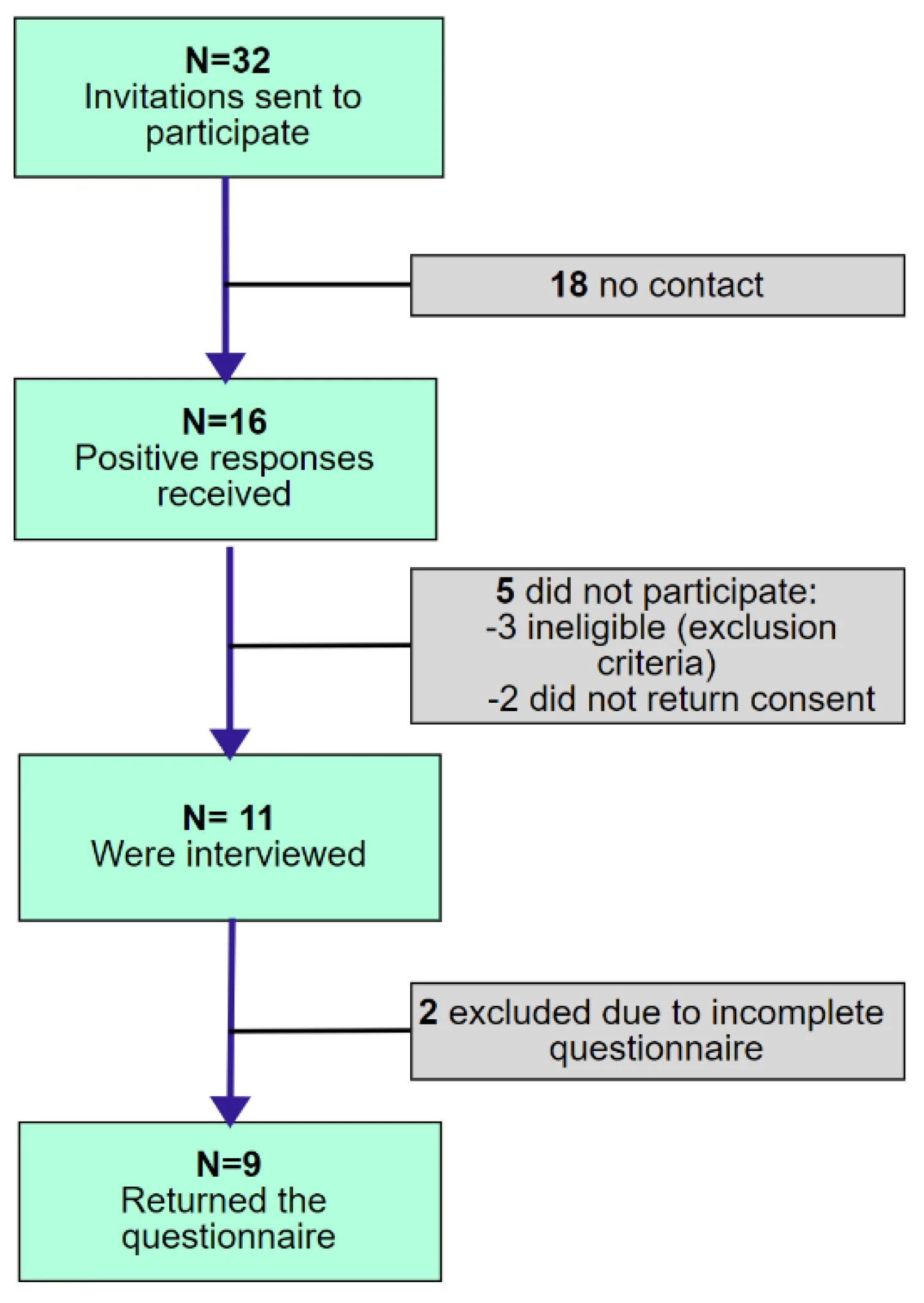
The flowchart of participant enrollment.

The participants were elite athletes who had suffered spinal cord injuries. The inclusion criteria adopted for the study were: informed consent to participate in the study, spinal cord injury (paraplegia or quadriplegia) and having won a medal in a sporting event of national or international rank before the SCI. For unhindered communication to be possible, all the participants also had to speak either Polish or English. Before starting the project, we analyzed information from media and national sports committees about elite athletes that had suffered SCIs in the last 20 years and selected 32 potential candidates who met the inclusion criteria from the following countries: Australia, Japan, Austria, Poland, UK, South Africa, USA, Canada and Brazil. They were invited by email, which contained a project description and the rules for the implementation of the survey. The participants’ inclusion process in the study is shown in Figure 1. The athletes who responded positively to the invitation were interviewed online prior to the study to verify criterium eligibility and to schedule an interview and cognitive function testing.

Sociodemographic information related to the participants is presented in Table 1. Before the spinal cord injury, all participants had won a medal at least on the national level, with five of them having been World or European champions. After their SCI, most of them had continued their career in sports in another discipline according to their functional capacity. Six athletes were competing in national and international competitions and two of them became Paralympic champions.

Table 2 shows health information about the study participants. They were selected among both tetraplegic and diplegic patients. Two of them had SCIs above the 4th cervical vertebra with diaphragmatic paralysis resulting in respiratory disorders during sleep (hypopnea and apnea). The main medications taken by patients after SCI were analgesics, antispastic, antidepressants and those used in neurogenic disorders, e.g., neurogenic bladder. In Table 2, the total number of drugs taken belonging to these groups is given.

### 2.2. Procedure

The athletes who participated in the study were interviewed via an online communicator. After the interview, they were asked to fill out an online personal survey and the WHOQoL-BREF (abbreviated version of the World Health Organization Quality of Life Scale). Prior to the interview, the participants were introduced to a consent form which contained all the detailed information about the project, including purpose, procedures and practical use of the collected data. The interviewer also presented the confidentiality rules; all athletes gave informed verbal consent for participation in the study and publication of the results in a way that allows them to be identified.

The first author, who has clinical experience and has been working for 12 years with patients after SCIs, conducted all the interviews. At the end of the interview, the second author, who is a clinical psychologist with 21 years of experience, conducted the following tests to assess cognitive functions: COWAT (Controlled Oral Word Association Test), Digit-Span and Stroop color–word. Each interview lasted approximately 1.5–2.5 h, was recorded and then transcribed. In the next step, the content was used to analyze and interpret the results. The plan of the research project was accepted by the Senate Research Ethics Committee of the Wroclaw University of Health and Sports Sciences, Poland (corresponding ethical approval code: 37/2018; art.27, Dz.U.1997, poz.553).

#### 2.2.1. Demographics and Injury Characteristics 

Sociodemographic data were collected using a personal questionnaire. In addition to personal information, the questionnaire included questions about athletic performance, information regarding spinal cord injury (level and time of injury) and factors that may influence the occurrence of CD, such as coexisting brain injury or whiplash, sleep-disordered breathing (hypopnea or apnea) and medications taken. Pain experienced daily was assessed using the 0–10 Numerical Rating Scale of Pain (0 = no pain, 10 = most intense pain).

#### 2.2.2. Quality of Life

In order to assess the quality of life, the abbreviated version of the WHOQoL with national adaptation scales was used. There are investigations confirming this scale is recognized as the appropriate instrument for assessing the QoL of people with SCIs [23,24]. 

The WHOQoL BREF is composed of questions describing 4 domains (physical health—D1; psychological—D2; social—D3; and material aspect—D4). Additionally, it gives information about subjective overall perception of the quality of life and physical health satisfaction [25,26]. 

The participants were requested to answer the questions using a five-level Likert scale as follows: agree (5—strongly agree; 4—agree), neutral (3—neither agree nor disagree), or dissatisfied (2—disagree; 1—strongly disagree). 

The collected raw point values were recalculated in each aspect of the WHOQoL-BREF scale on a scoring scale ranging from 4 to 20, according to the WHO recommendations [25]. A high score value indicated a high participant’s quality of life [26]. The Cronbach’s alpha coefficients for WHOQOL-BREF domains were: D1 α = 0.76; D2 α = 0.81; D3 α = 0.71; and D4 α = 0.9 [25].

#### 2.2.3. Cognitive Performance

##### COWAT

Phonemic verbal fluency was assessed using the COWAT tool [27]. This test is one of the basic methods of neurocognitive assessment; during the test, the functions of speech, memory and executive processes are involved. The method provides an index of mental productivity, language processes and the functioning of, primarily, the frontal and temporal lobes [27,28]. The participant of the study was asked to give oral associations of different letters of the alphabet, saying all the words that can start with a given letter within 60 s. Three letters, F, A and S, with progressively increasing levels of associative difficulty, were sequentially presented as stimuli. The difficulty level of each letter was defined as the relative frequency of words beginning with that letter. Responses were recorded, words were transcribed and then counted. The standard score was quantitative and included the number of words matching the given criterion and errors, i.e., out-of-category responses and repetitions. The internal consistency of COWAT was α = 0.83 [29]. 

##### Digit Span Test

The Digit Span test consists of two parts, a digits forward test (DForw) and a digits backward test (DBack). DForw captures the efficiency and capacity of attention. The DBack test is a performance task that is particularly dependent on working memory [2,9]. In the DForw test, participants were asked to repeat, in the same order, a series of random digits that were read to them. The initial length included two digits. After successfully completing a given trial by repeating six digits with up to one error, the participant was presented with the next pair with a single digit increment. The test ended when the participant misrepresented numbers on more than one trial with the same span or successfully repeated five nine-digit sequences. The recorded score (DForw) was the number of sequences correctly recalled. The DBack test has the same procedure as the DForw test except that patients repeated the digits in reverse order. The DBack score was the number of correct reverse sequences. The Cronbach’s alpha coefficient for the test was α = 0.74 [3].

##### Stroop Color–Word Test

Cognitive interaction inhibition ability, attention, cognitive flexibility, processing speed and verbal and working memory were examined using the Stroop color–word test. Three pages of the test were sent to the participant and were sequentially displayed by them on a computer screen during the test. In the word-reading trial, the tested person read as quickly as possible the words denoting the names of colors written in black print on a white sheet of paper in 10 rows of 5 words each. In the color-reading trial, participants read the print color of the individual “xxxx” sequences. In the word–color (interference) trial, they named the print color of individual words with the print color not matching its designator. The test score represented the number of words read within 45 s in each part of the test. The Cronbach’s alpha value of the Stroop color–word test ranged from 0.71 to 0.88 [30].

### 2.3. Data Analysis

To the best of our knowledge, the present study was the first attempt to analyze the quality of life and cognitive function of individuals after SCIs. Therefore, we calculated the mean and standard deviation of questions Q1 and Q2 and the individual domains of WHOQoL-BREF, as well as individual tests assessing cognitive function (COWAT, Digit-Span test and Stroop color–word test). To assess the relationships between the included variables, Spearman’s rank correlations were used, with *p* < 0.05 indicating statistically significant results. In the following procedure, in order to exclude the occurrence of false statistically significant results, in view of the large number of correlations, a correction Bonferroni method was applied. In addition, using the alpha correction method, the minimum group size required to detect a moderate (0.3) and a large effect size (0.5) was determined. All the analyses were performed in Statistica (ver. 13.1) at the Biostructure Research Laboratory of Wroclaw University of Health and Sport Sciences (certificate ISO 9001).

## 3. Results

### 3.1. Quality of Life

Table 3 shows the participants’ scores in two general items (Q1—overall perception of QoL; Q2—health satisfaction) and four domains (D1—physical health; D2—psychological; D3—social; D4—material aspect), along with the mean and standard deviation. The mean value for Q1 was 4.11, which indicates a positive assessment of overall quality of life (results >3). Only two participants (P1 and P5) rated their quality of life negatively (results ≤3); these two individuals were also not satisfied with their health status. In a comment on the WHOQoL-BREF scale, participant P1 indicated that he had been in poor health for the past 4 weeks, which is the period referred to by the scale questions and it had the effect of impairing both physical and mental well-being, as well as social isolation, with the need to stay in bed. The lowest scores in all domains of quality of life were obtained by participant P5, who indicated a depressive personality, which worsened after the SCI.

### 3.2. Cognitive Performance

Table 4 shows the participants’ scores on tests assessing cognitive functions, COWAT, Digit Span and Stroop color–word, along with the mean and standard deviation. The lowest scores on the COWAT were obtained by P1 and P9, while both Digit Span and Stroop color–word tests were obtained by participant 4. In contrast, the highest scores on all cognitive tests were obtained by P8.

Spearman’s correlation showed that overall satisfaction with physical health and the physical domain was related to the Stroop color–word subtest and pain was related to one of the subtests of the COWAT (Table 5). The Bonferroni correction reduced the significance level to α = 0.0007. Therefore, it should be assumed that no statistically significant relationships between the studied variables were demonstrated, but this is not equivalent to a conclusion that these relationships would not be observed in a larger group of participants. After applying the alpha correction, it was determined that the minimum sample size for at least a medium effect (0.3) should be *n* = 19. 

## 4. Discussion

The study presents the quality of life and cognitive functioning of nine top athletes after SCIs and, at the same time, attempts to search for the correlations between these measures. To date, there has been research on the relationship between quality of life and cognitive function in many disease entities, but, to the best of our knowledge, this study is the first to assess this aspect among population of patients with SCIs, besides analyzingelite athletes.

Despite the traumatic spinal cord injury and the many years passed since the accident (>4 years), the study participants generally described their quality of life as positive. Only two participants rated their quality of life negatively (scores ≤ 3), which may be explained by P1’s poor health over the past 4 weeks and P5’s depressive personality. P5 was also characterized by the shortest time elapsed since the accident. Saunders et al. (2012), in their study among 801 adults after SCIs, indicated that increased time since injury is a protective factor in the occurrence of major depressive disorders [31]. A similar finding was observed in this study, as the participants accepted their disability and had adapted to their new living situation because of the time that had passed since the accident. Six participants had changed their sport and were still physically active, while the others participated in sports life as coaches and motivational speakers. This may explain the high scores in the psychological, social and environmental domains of the quality-of-life scale. In addition, Byra et al. (2019), in their study, indicated a positive correlation among disability acceptance, post-traumatic growth, an enhancement of mental health and social adaptation in those who were positive about disability [32,33]. It is also worth noting that the majority of participants (*n* = 6), despite having biplegia or quadriplegia, were generally satisfied with their health. P5, despite having a better functional condition than others, rated his physical health negatively due to severe pain and depressive personality. This confirms the results of previous studies that concluded that it is the perceived physicality that has been lost as a result of the injury—and not an objective physical limitation—that determines satisfaction with one’s health [34]. Previous studies also indicated that health satisfaction was influenced more by secondary health issues than by primary accident-related damage, which is also indicated by the results of our study [35,36,37].

In the COWAT, according to normative data by age, all participants except P8 scored significantly below the average for their age category, which indicates decreased memory functions and executive processes [38]. Among the athletes, the lowest scores were obtained by P1 and P9. Of the factors that may affect cognitive dysfunction, P1 was characterized by breathing disorders while sleeping, coexisting brain injury during the accident, severe pain and the highest number of medications taken (eight medications). In contrast, P9 experienced severe pain while not taking any pain medication, which may explain his low score on the COWAT, compared with the other participants. Comparing the results of the respondents in cognitive tests with available studies by age in the Digit Span test, only P4 ranked significantly below the average for their age category and P7 and P9 in the backward subtest for their age category [39]. The rest of the participants ranked above average, indicating normal performance and capacity of attention and working memory. The lowest scores in both the Digit Span test and Stroop color–word test were obtained by P4, which may be due to high spinal cord injury at the C4/5 level, occurrence of respiratory distress during sleep and coexistence of whiplash during the accident. The highest scores in all tests were obtained by P8, in whom no potential causes of cognitive impairment were observed. P8 also rated his quality of life and his physical health positively. At the same time, P8 trained canoe professionally and, while performing the cognitive tests, he was also in the process of preparing for the Paralympics. Thus, high scores in cognitive tests may be a result of physical activity, which increases the level of circulating BDNF protein, which has neuroprotective and neurotrophic effects and positively influences cognitive processes [40].

Although the research study conducted indicated that some cognitive functions decreased among athletes, the study did not show a statistically significant correlation with decreased QoL. However, the fact that there was no significant correlation between the included variables in this study should be treated with great caution due to the small group of participants. It is also important to note that, to date, there has been no study of individuals with SCIs that has examined the relationship between cognitive functions and QoL; therefore, there is no reference point for conducting larger analyses in other, larger groups of participants. Considering this limitation, it is important to note that this occurrence would not necessarily be absent if similar studies were to be conducted on a larger sample. Furthermore, we are dealing with outstanding athletes where specific external and internal factors could be considered as determinants affecting the results. As elite sportsmen, they had definitely more opportunities to benefit from, such as various types of social support, wide interest from media and friends, continuous rehabilitation and the possibility of frequent leaving home, which eventually results into the fact of participation in social life without exclusion. In a study by Wang et al. (2016), 120 patients with migraine-associated vertigo showed an increased prevalence of white matter lesions in the brain, resulting in marked cognitive impairment and, consequently, a significantly impaired quality of life [17]. In addition, a study by Molina-Gallego et al. (2021) on a group of 100 participants showed that decreased cognitive functioning could negatively affect the quality of life and social interactions, but it was not observed in our study [14]. The athletes were characterized by the long time that had passed since the accident. This may be the reason for the lack of statistically significant correlation between cognitive level and QoL, as respondents had already passed various linear stages of adjustment, leading to optimal adjustment to SCI. SCI patients receive much less professional support and assistance after hospital discharge and their cognitive, emotional, social and mental resources that are necessary for independent coping and adjustment are challenged [4]. On the other hand, Lanzillo et al. (2016), in a study involving 54 people with multiple sclerosis, showed that cognitive decline was mainly influenced by physical disability and poor social engagement, concluding that affective relations and psychological flexibility could have a protective function on CD [19]. The participants, despite the long time that had passed since the injury, participated in sport life in both active (sport for people with disabilities) and passive (role of coach, motivational speeches) forms, which, despite the deterioration of some cognitive functions, allows them to maintain affective relations. High social support in elite athletes may influence the lack of statistical significant relationship between reduced level of some cognitive functions and positively rated quality of life. A protective factor may also be high emotional intelligence, which consists of personal competencies (self-awareness, self-regulation and motivation) and social competencies, characterized by athletes with high sporting achievements [41]. A strong personality can help to continue to function after SCIs and cause greater resilience to the circumstances despite starting a changed life [42].

The analysis of the present study was conditioned by the limitations resulting from the specific study group of elite athletes with SCI. One of them is the size of the study group, which was due to the prepared inclusion criteria. Although a twenty-year period was considered and cases of SCIs in outstanding athletes were analyzed, nine such individuals were ultimately able to be included in the project. The authors realize that the small number of subjects is a limitation, but this is the first study of quality of life and cognitive functions among people after SCI, as well as among outstanding athletes after SCI. Therefore, the results of the study can be regarded as valuable and interesting material for further comparisons. However, it should be kept in mind that the study participants, as individuals with outstanding athletic achievements, were characterized by special conditions that may be relevant to the final interpretation of their results. Although the study shows the relationships between the included variables, it can be seen as a starting point for further, more extensive research. Secondly, due to the fact that they lived in different continents, the only possible form of conducting this type of study was remote. Thirdly, the form of the study had to be tailored to the participant with the least functional abilities, thus with the complete exclusion of the use of upper limbs, which significantly limited the possibility of using tests assessing cognitive performance. This limiting factor has also been pointed out by Pasipanoydia et al. (2021), highlighting the need for development and validation of neuropsychological test batteries for screening and assessment among patients with limited motor abilities [15]. Factors that were not considered may also contribute to CD, such as alcoholism, substance abuse, previous learning problems, or emotional problems. Although all participants were outstanding athletes, this group could not be treated as homogeneous due to the different information collected in the study. The consequence of the above is the difficulty in obtaining a result allowing unambiguous conclusions to be drawn and generalized to the entire population.

Despite these limitation, this study is the first to determine the relationship between QoL and cognitive disorders among SCI patients, as well as in a group of elite athletes. Given the inextricable link between cognitive status and QoL, in view of the inconclusive results, further studies involving a larger number of participants with SCI are indicated to help inform and guide interventional approaches. A larger number of participants would allow a broader analysis of the results in subgroups to be performed, such as level of SCI, pain, respiratory distress and medications taken. The introduction of a control group of non-athletic SCI patients is also an option for future studies.

## 5. Conclusions

Despite the traumatic accident, the participants in this study positively evaluated their health and quality of life. The careful analysis of the results obtained by the athletes suggested that their reduced cognitive functioning could have been influenced by the impact of sleep-disordered breathing, pain, depressive disorders and medication. This underscores the need to treat each patient individually to diagnose their problems and the reasons underlying them.

## Figures and Tables

**Table 1:** Sociodemographic data of study participants.

Participant	Age	Gender	Nationality	Marital Status	Discipline before SCI	Sport after SCI
P1	41	Male	British	Divorced	BMX dirt jumps	No
P2	29	Male	Austrian	Single	Ski jumping	Rugby, skiing
P3	24	Female	Polish	Informal relation	Karate	Wheelchair dancing
P4	37	Male	British	Informal relation	Rugby	No
P5	55	Male	American	Married	Mountain bike racing	No
P6	45	Female	Canadian	Married	Mountain biking	Wheelchair basketball
P7	31	Male	British	Informal relation	Motocross	Car race
P8	40	Male	Polish	Informal relation	Judo	Canoe
P9	47	Male	Polish	Single	Speedway	Hand cycling

**Table 2:** Health data of the participants.

Participant	SCI Level	Years since Injury	Pain	Brain Injury/Whiplash	Hypopnea/Apnea	Medicines (Number)
P1	C3/4	14	7	B	Yes	8
P2	C6/7	5	7	W	No	1
P3	Th11/12	6	3	No	No	0
P4	C4/5	16	0	W	Yes	1
P5	C6/7	4	6	No	No	3
P6	Th12/L1	14	1	W	No	1
P7	Th6	15	0	B	No	2
P8	Th11	17	3	No	No	0
P9	L1/2	15	7	No	No	0

**Table 3:** WHOQoL-BREF scores (* lowest scale scores).

Scale		P1	P2	P3	P4	P5	P6	P7	P8	P9	Mean ± SD
WHOQOL	Q1	3 *	4	4	5	2 *	5	5	4	5	4.1 ± 1.1
	Q2	1 *	5	4	3	2 *	5	5	4	5	3.8 ± 1.5
	D1	14	18	11 *	15	11 *	19	20	16	14	15.3 ± 3.2
	D2	13 *	19	15	15	7 *	19	17	15	20	15.6 ± 4.0
	D3	13 *	20	12 *	17	9 *	19	17	16	20	15.9 ± 3.8
	D4	16	17	16	17	13 *	20	20	13 *	20	16.9 ± 2.8

**Table 4:** COWAT, Digit Span (DForw and DBack) test and Stroop color–word test—correct answers (* lowest scale scores).

Scale		P1	P2	P3	P4	P5	P6	P7	P8	P9	Mean ± SD
COWAT	F	6 *	12	9	11	11	9	10	12	6 *	10.0 ± 2.0
	A	6 *	8	8	9	10	15	11	18	6 *	10.0 ± 4.0
	S	6 *	11	17	7	9	7	13	19	9	11.0 ± 4.6
	All	18 *	31	34	27	30	31	34	49	21 *	31.0 ± 8.8
Digit Span	DForw	7	7	6 *	3 *	7	7	7	8	5	6.0 ± 2.0
	DBack	4	6	4	3 *	4	4	2 *	6	2 *	4.0 ± 1.0
Stroop color– word test	Word correct answers	92	90	86	54 *	77	97	80	127	82	87.0 ± 19.0
	Color correct answers	54	58	42 *	42 *	60	76	71	75	70	61.0 ± 13.0
	Word and color correct answers	36	53	46	36	31 *	51	60	53	52	46.0 ± 10.0

**Table 5:** Spearman’s rank order correlation.

		COWAT				Digit Span		Stroop		
		F	A	S	All	DForw	DBack	Word	Color	Word–Color
WHOQoL	Q1	−0.18	0.17	−0.05	0.07	−0.44	−0.60	−0.16	0.29	0.46
	Q2	0.03	0.17	0.34	0.37	0.00	−0.17	0.17	0.53	0.79
	D1	0.28	0.51	0.08	0.37	0.36	−0.02	0.27	0.59	0.73
	D2	−0.14	−0.07	0.10	0.08	−0.21	−0.23	0.17	0.40	0.67
	D3	−0.01	−0.12	−0.11	−0.12	−0.19	−0.17	0.12	0.32	0.58
	D4	−0.39	−0.11	−0.25	−0.16	−0.39	−0.62	−0.14	0.28	0.44
Pain		−0.21	−0.67	−0.13	−0.46	0.10	0.33	0.27	−0.15	−0.12
Medicines		−0.09	−0.07	−0.57	−0.43	0.23	−0.09	−0.24	−0.10	−0.37

## Data Availability

The datasets used and/or analyzed during this study are available from the corresponding author upon reasonable request.
